# Comparing the DSM-5 construct of Disruptive Mood Dysregulation Disorder and ICD-10 Mixed Disorder of Emotion and Conduct in the UK Longitudinal Assessment of Manic Symptoms (UK-LAMS) Study

**DOI:** 10.1007/s00787-018-1149-5

**Published:** 2018-05-05

**Authors:** I. Sagar-Ouriaghli, G. Milavic, R. Barton, N. Heaney, F. Fiori, K. Lievesley, J. Singh, Paramala Santosh

**Affiliations:** 1 Department of Child and Adolescent Psychiatry, King’s College London, London, UK; 2 Centre for Interventional Paediatric Psychopharmacology and Rare Diseases (CIPPRD) Research Team, South London and Maudsley NHS Foundation Trust, 4 Windsor Walk, Denmark Hill, London, SE5 8BB UK; 3 South London and Maudsley NHS Foundation Trust, London, UK

**Keywords:** Disruptive Mood Dysregulation Disorder, Mixed Disorder of Emotion and Conduct, Longitudinal Assessment of Manic Symptoms, LAMS, DSM-5, ICD-10

## Abstract

It is important to understand new diagnostic entities in classifications of psychopathology such as the Diagnostic and Statistical Manual of Mental Disorders-5 (DSM-5) (code F34.8) construct of Disruptive Mood Dysregulation Disorder (DMDD) and to compare it with possible equivalent disorders in other classificatory systems such as the International Classification of Diseases-10 (ICD-10), which has a category that superficially appears similar, that is, Mixed Disorder of Emotion and Conduct (MDEC) (code F92). In this study, the United Kingdom (UK) arm (UK-LAMS) of the US National Institute of Mental Health (NIMH) supported Longitudinal Assessment of Manic Symptoms (LAMS) multi-site study was used to evaluate and retrospectively construct DMDD and MDEC diagnoses in order to compare them and understand the conditions they co-occur with, in order to improve the clinical understanding. In particular, the phenomenology of UK-LAMS participants (*n* = 117) was used to determine whether DMDD is a unique entity within the DSM-5. The findings showed that 24 of 68 participants with either DMDD or MDEC (35.3%) fulfilled both diagnostic criteria for DMDD and MDEC, suggesting that these entities do contain overlapping features, particularly symptoms relating to Oppositional Defiant Disorder (ODD)/Conduct Disorder (CD), Attention Deficit Hyperactivity Disorder (ADHD)/Hyperkinetic Disorder (HKD) and/or an anxiety disorder. The data also showed that most of the participants who met DMDD criteria also fulfilled the diagnostic criteria for ODD/CD, ADHD, followed by an anxiety disorder. In this context, this raises the issue whether DMDD is a unique construct or whether the symptomology for DMDD can be better explained as a specifier for ODD/CD and ADHD. Unlike DMDD, MDEC clearly specifies that the label should only be used if emotional and conduct disorders co-exist.

## Introduction

Mood dysregulation is a very common presenting feature in multiple psychiatric disorders; for example, it can be seen in Attention Deficit Hyperactivity Disorder (ADHD), Autism Spectrum Disorders, Oppositional Defiant Disorder (ODD), Tourette’s syndrome, Obsessive Compulsive Disorder (OCD), Anxiety Disorders, Depression, Bipolar Disorders (BD), amongst others. In the recent past, there was a tendency to label many such individuals as having Paediatric Bipolar Disorder (PBD), leading to a significant increase in numbers being given the diagnosis. This led to re-examining what label should be given to such individuals instead. Severe Mood Dysregulation (SMD) disorder was constructed to help operationalise severe irritability [1], defined as having two components: frequent and extreme inappropriate temper outbursts in accordance with developmental age and the presence of negative mood, such as anger or sadness, between these outbursts [2]. Unlike PBD, SMD is regarded as non-episodic and symptoms of psychosis or brief manic or hypomanic episodes are not present in SMD [2]. Evidence suggests that children with SMD are able to modulate their attention properly in the context of increased emotional demands compared to children with PBD [3]. Differences in event-related potential during frustration have been identified, with decreased activity from parietal sites (P3) in PBD patients compared to normal P3 activity and decreased event-related potential (N1) in SMD patients [3]. Variations in P3 and N1 activity between those with PBD and SMD provide further explanation for affective symptoms, where P3 deficits (identified in PBD) have been associated with depression [4] and N1 deficits, seen in SMD, have been linked to ODD symptom severity [3]. From ongoing debate and existing research, SMD was then formalized as Disruptive Mood Dysregulation Disorder (DMDD) in the Diagnostic and Statistical Manual of Mental Disorders-5 (DSM-5). Hence, both SMD and DMDD share similar diagnostic criteria, with the exception of SMD requiring to fulfil at least three domains of hyperarousal (insomnia, agitation, distractibility, racing thoughts or flight of ideas, pressured speech and intrusiveness [2]) and that DMDD cannot co-exist with ODD [5], where SMD can. It was clearly developed as a diagnosis that was not related to the Bipolar Disorder phenotype.

DMDD is characterised as extreme episodes of irritability, anger with frequent and intense temper outbursts in children. In lay terms, DMDD symptoms extend beyond a child that is moody, resulting in chronic impairment requiring significant clinical management. In order to meet the criteria for DMDD, the symptoms of irritability/anger are experienced most of the day nearly every day, with severe temper outburst roughly three or more times a week (outside of the child’s developmental level), where these difficulties are present in more than one setting. In order to fulfil a diagnosis a child must have these symptoms consistently across a minimum of 12 months [5, 6].

DMDD is not without its limitations and although previously it was viewed as a diagnostic home for children with severe chronic irritability that do not meet the criteria for PBD, in practical terms the diagnosis of DMDD remains controversial [7]. Irritability is a broad construct in typically developing youth [8], however, in recent years the clear distinction between PBD and DMDD centres on clear and distinct episodes of irritability within PBD [9].

DMDD attracts controversy as it frequently presents in several other psychopathologies [10–12]. In some instances, the diagnosis of DMDD only contributes to diagnostic confusion, whereby it has been suggested that the phrase ‘with irritable mood’ should be added to the ODD diagnosis [13, 14]. ODD has been shown to explain the majority of the variance in DMDD symptoms and can enable the prediction of ODD symptoms [15]. Furthermore, DMDD has been shown to operate in the context of other disorders [16] and is significantly associated with co-occurring diagnosis of anxiety, depression, ADHD and ODD [7, 17]. From a longitudinal perspective, DMDD shows a limited diagnostic stability over time and cannot be demarcated reliably from childhood disorders such as ODD and Conduct Disorder (CD) and that for the US Longitudinal Assessment of Manic Symptoms (LAMS) cohort, DMDD was not associated with mood or anxiety disorders irrespective of current or future onset [18].

As the DSM-5 is not the only major classificatory system used, we sought to identify if these findings from the DSM carry over to other manuals with similar constructs such as those within the International Classification of Diseases-10 (ICD-10) [19]. To date, there is very limited literature surrounding the ICD-10 construct of Mixed Disorder of Emotions and Conduct (MDEC, code F92), which is commonly used by clinicians when dealing with symptoms such as those seen in DMDD. In accordance with the ICD-10, MDEC is classified as a group of disorders presenting with the combination of persistent aggression, dissocial or defiant behaviour, in conjunction with overt symptoms of depression, anxiety or other emotional upsets.

In lay terms, this maps closely to the DMDD diagnosis and captures those children who present with chronic irritability and threshold symptoms for various anxiety disorders (social anxiety, phobic anxiety, separation anxiety generalised anxiety, or OCD), or a mood disorder (mania, hypomania, bipolar disorder, or a depressive disorder).

Due to this, the current report sought to investigate MDEC in greater detail in order to see if it can be mapped to the DMDD criteria on a symptom level, as this would allow for a clearer understanding as to whether the MDEC and DMDD are indeed similar, or unique constructs for their respective manuals. Furthermore, much of the current literature places great emphasis on DMDD and has been extensively explored in the US arm of the LAMS study, suggesting that rather than DMDD posing as a separate diagnostic category, it may be better classified as a specifier for ODD [18]. In light of this, we sought to explore MDEC in a similar manner to see if it too may be better explained as a specifier for ODD, as opposed to a separate diagnostic category. Lastly, in addition to these main areas the current report also sought to explore the co-occurring disorders within DMDD and MDEC.

Due to its large sample size and clinical utility, the United Kingdom (UK) arm (UK-LAMS) of the US NIMH supported LAMS multi-site study was used, where one is able to evaluate and retrospectively construct DMDD and MDEC diagnoses in order to examine clinically meaningful issues.

## Methods

### Study participants

The US version of LAMS was a prospective study that recruited youth aged 6–12 years of age with elevated symptoms of mania from 4 academic medical centers (Case Western Reserve University, Cincinnati Children’s Medical Center, Ohio State University, and the University of Pittsburgh Medical Center) and a detailed description of the LAMS study design and methodology has previously been described elsewhere [20]. The UK cohort was recruited at the National and Specialist Depression and Bipolar Disorder Clinic, Maudsley Hospital, UK. In brief, the objective of the UK-LAMS was to follow a group of children aged 6–12 years old with elevated symptoms of mania (labelled ESM+) longitudinally and compare them with a group of children without ESM (labelled ESM−). Further, it is aimed to define the characteristics of ESM+ children and establish whether a relationship existed between ESM identified manic symptoms and BD, and risk of developing BD.

### Participant recruitment

Recruitment information for the LAMS study is described in Horwitz et al. [20]. In brief, for UK-LAMS, parents/carers of children who scored their child at or above a score of 12 (i.e. ESM+) on the Parent General Behavior Inventory 10-Item Mania Scale (PGBI-10M) were invited to participate in the study. Likewise, matched participants who rated ≤ 11 (i.e. ESM−) were also enrolled. The inclusion criteria for UK-LAMS were (1) child is aged 6–12 years old, (2) child is accompanied by a parent/carer, (3) family are English speaking, (4) another household member has/is not enrolled in LAMS, (5) child has not been previously approached to participate in LAMS and (6) child does not have a diagnosis of Autism (Pervasive Developmental Disorder-Not Otherwise Specified [PDD-NOS]) and Asperger’s were accepted.

### UK-LAMS: retrospective DMDD diagnosis

Items from the Schedule for Affective Disorders and Schizophrenia adapted for school age children (KSADS) were used to identify those who would have met the diagnoses of DMDD (DSM-5, code F34.8), and/or MDEC (ICD-10, code F92). The descriptions of the filters (1-5) used to match DMDD are shown in Appendix A.

### UK-LAMS: MDEC diagnosis

Items were used from the Schedule for Affective Disorders and Schizophrenia adapted for school age children (KSADS), and the Child and Adolescent Symptom Inventory-4 (CAASI-4) scale to make the diagnosis of MDEC according to the ICD-10 (code F92). The descriptions of the filters (1–7) used to make the diagnosis of MDEC are shown in Appendix B.

Despite both MDEC and DMDD capturing similar presenting symptoms, it is to be noted that an MDEC diagnosis can only be given if the criteria for CD of childhood are met. The KSADS was used predominately for the purpose of the current report as it has been shown to be a valid tool for retrospective diagnoses [21] with good convergent and adequate divergent validity [22, 23] and is suitable at detecting high comorbidity in children and adolescents [24]. Specifically, the exclusion criteria of comorbid disorders for both DMDD and MDEC were ignored, as we were interested in identifying co-occurring disorders.

## Results

From the data obtained on a total of 117 individuals (including both ESM+ and ESM−), 68 individuals fulfilled either the DSM-5 criteria for DMDD (*n* = 47) or the ICD-10 criteria for MDEC (*n* = 45). Following this, descriptive frequencies of other K-SADS-PL diagnoses within the sample are summarised in Table 1.

**Table 1:** Frequencies of other diagnoses in both the DMDD and MDEC participants

KSADS diagnosis (*n *= 117)	Frequency of diagnosis within entire sample (*n* = 117)	For those who meet DMDD criteria (*n* = 47)	For those who meet MDEC criteria (*n* = 45)	For those who meet both DMDD and MDEC criteria (*n* = 24)
Bipolar disorder	7	7	2	2
Depressive disorder	15	10	8	6
Anxiety disorder	53	26	32	18
ADHD	87	41	36	19
ODD/CD	74	43	38	23
PDD (including ASD)	26	12	10	6

*ADHD* Attention Deficit Hyperactivity Disorder, *CD* Conduct Disorder, *DMDD* Disruptive Mood Dysregulation Disorder, *MDEC* Mixed Disorder of Emotion and Conduct, *ODD* Oppositional Defiant Disorder, *PDD* pervasive developmental disorder

Within the total sample, 47 individuals met the criteria for DMDD and 45 met the criteria for MDEC. As expected, none of those who fulfil DMDD or MDEC criteria have a sole diagnosis of ODD or CD. From both sets of patients, the concordance between the two criteria was estimated by identifying the frequency of those meeting both DMDD and MDEC criteria. From these results, 24 (35.29%) patients were identified as fulfilling both DMDD and MDEC criteria.

Considering these points highlighted above, further investigation of the data sought to highlight the overlap of the DMDD and MDEC with other diagnostic labels, with focus on ODD/CD, anxiety disorders, and ADHD, and ICD-10 diagnosis of Hyperkinetic disorder (HKD). Table 2 presents a breakdown of the overlapping diagnoses for the participants who had DMDD and MDEC.

**Table 2:** Frequencies and percentages of those who meet comorbid diagnosis from the DMDD and MDEC participants

Co-occurring disorders^a^	Frequency	% of DMDD sample
DMDD	47	100.0
ODD/CD + ADHD	18	38.3
ODD/CD + ADHD + anxiety disorder	18	38.3
ODD/CD + anxiety disorder	6	12.8
ADHD	3	6.4
ADHD + anxiety disorder	2	4.3

Co-occurring disorders	Frequency	% of MDEC sample
MDEC	45	100.0
ODD/CD + anxiety disorder + HKD or ADHD	22	48.9
ODD/CD + HKD or ADHD	8	17.7
ODD/CD + anxiety disorder	7	15.6
ADHD or HKD	5	11.1
Anxiety disorder	2	4.4
Anxiety disorder + HKD	1	2.2

*ADHD* Attention Deficit Hyperactivity Disorder, *CD* Conduct Disorder, *DMDD* Disruptive Mood Dysregulation Disorder, *HKD* Hyperkinetic Disorder, *MDEC* Mixed Disorder of Emotion and Conduct, *OCD* Obsessive Compulsive Disorder, *ODD* Oppositional Defiant Disorder, *TR* text revision

^a^Co-occurring disorders were based on DSM-IV-TR with the exception of HKD, which was determined by ICD-10

A Pearson’s Chi square test was conducted between those who met both MDEC and DMDD in order to estimate the concordance rates of those fulfilling both the MDEC and DMDD criteria. Results showed a significant difference between the two diagnostic criteria (*χ*^2^ = 4.544; Fisher’s exact test, *p* = .036).

## Discussion

The primary focus of the current study was to investigate as to whether the construct of DMDD and MDEC overlap with one another. From the data presented, despite some subjects meeting both criteria, there was a significant difference between these two constructs, thus, indicating that they can be seen as relatively independent constructs. The data presented show that for subjects with DMDD or MDEC, 50% or more participants also met the criteria for of ODD/CD, with an anxiety disorder and/or ADHD/HKD, indicating that they do capture similar psychiatric symptoms.

As highlighted in the previous literature, most of the participants who met DMDD also fulfilled the diagnostic criteria for ODD/CD with ADHD [18, 25] followed by an anxiety disorder [26]. Within the current paper, we have highlighted that the symptomology for DMDD can be better explained as a specifier of ODD/CD and ADHD. Similar to the entire LAMS study [18], a significant number of participants in the UK-LAMS arm fulfilled DMDD and/or MDEC diagnoses (58.12%). Previously, Axelson et al. [18] have explored the DMDD construct in greater detail noting that DMDD was only specifically associated with ODD and CD with 40–50% of youth also presenting with anxiety, depression and bipolar disorder. Our current findings re-iterate the association with ODD/CD but also place greater emphasis on an anxiety disorder as well as ADHD. It is apparent that this is a common theme across the DMDD literature; however, the interaction between ODD/CD, anxiety and ADHD in the context of DMDD is yet to be explored. Our current knowledge of DMDD seems to point towards it being a specifier for particular disorders, with these findings contributing to this notion. Unlike DMDD, the ICD-10 MDEC diagnosis specifies that the label should only be used if emotional and conduct disorders co-exist. We demonstrate that MDEC is mainly being diagnosed when ODD/CD is co-occurring with at least one anxiety disorder and/or ADHD/HKD. Surprisingly, it did not present with a frequent diagnosis of depression.

Both the DMDD and MDEC constructs appear to be alternative ways of describing the presence of ODD/CD with either anxiety or ADHD/HKD. Indeed, one wonders whether an individual diagnostic label of DMDD adds any value other than that already provided by the diagnoses of ODD/CD, ADHD, and an anxiety disorder. Clinically, it is common to be dealing with emotional and behavioural dysregulation as core symptoms of multiple disorders, with recent evidence highlighting the consideration of autonomic dysregulation [27]. Both DMDD and MDEC have attempted to provide categories where these individuals can be classified, yet these categories seem to overlap with other diagnostic criteria, presenting more confusion surrounding the symptomology of individuals who present with these symptoms alongside other pre-existing diagnoses such as ODD/CD. Indeed this results in patients obtaining multiple labels, or a comorbid diagnosis (e.g. DMDD and ODD) as opposed to one diagnosis with a specific sub-type (e.g. ODD, DMDD specified).

By acknowledging that both DMDD and MDEC present as variants of different facets and diagnoses, one must consider what the best approach is for individuals who fulfil the diagnostic criteria of DMDD or MDEC. By adopting DMDD and MDEC as a specifier for the variations in symptom severity seen in ODD/CD, ADHD and anxiety disorders, it enables a more specific approach for each individual, which in turn may help to improve clinical communication and allow for a more detailed understanding of specific symptoms. This would hopefully be more informative to clinicians, allowing for faster and more accurate identification of treatable symptoms. Our findings question the added value of DMDD and MDEC for clinical purposes, as it may pose some risk of clinicians overlooking “treatable” diagnoses that are included within the mixed categories, such as ADHD/ODD/CD and anxiety disorders. Therefore, these labels may hinder rather than facilitate communication about diagnosis and treatment.

However, rather than clinical value, the labels of DMDD and MDEC may provide larger, more global benefits to the patient and the current healthcare infrastructure. For instance, this may lead to quicker identification of this patient group, inform future health care policy, improve access to care, and potentially highlight the need to involve multiple services and/or a larger multi-disciplinary team for each patient. Based on our findings, as the majority with DMDD and MDEC have a broad range of symptoms more commonly associated with ODD/CD, ADHD/HKD and anxiety disorders, assessing and monitoring these individuals and, targeting treatments to specific domains involved in each individual will be necessary to personalise care to optimise outcomes.

There is a fundamental problem with trying to conduct clinical trials for emotional dysregulation, as this is unlikely to be encouraged by the FDA or EMEA, as it would be a symptom cutting across multiple diagnoses. Therefore, focussing on a clinical trial in DMDD may be more prudent, rather than looking at emotional dysregulation.

The results highlighted should be considered on the backdrop of the following limitations. The KSADS screens for a range of psychiatric disorders and thus symptoms from the KSADS were utilised to determine DMDD and MDEC. Symptoms captured on standardised scales were used to identify those with DMDD and MDEC, rather than the diagnoses having been made prospectively and, therefore, has the potential to confound the number of those fulfilling both MDEC and DMDD criteria. Due to this issue, although ADHD, ODD/CD and anxiety all heavily overlap with MDEC/DMDD, this may be diagnosed at lower rates in clinical practice, where diagnostic instruments are not being used and only clinical criteria of the DSM and ICD are used. Furthermore, the KSADS diagnostic criteria are aligned with the DSM-IV-TR, which presents difficulties with mapping with DMDD, which is a DSM-5 construct. Finally, many of these participants were recruited from outpatient mental health services and, thus, the generalisation of findings might not be applicable to other mental health settings or services.

## Conclusion

The current paper highlights the confusion surrounding irritability and aggression in young children and explores the diagnostic descriptions for those with chronic irritability. Although DMDD has its limitations, it is still a unique entity from MDEC as defined by the ICD-10. The diagnostic label of DMDD and now MDEC both appear to capture individuals who fulfil diagnostic criteria for ODD/CD, ADHD and/or an anxiety disorder, all of which needs to be considered when managing them, along with the consideration that both DMDD and MDEC may be better viewed as specifiers.

## Appendices

### Appendix A: DMDD filters

#### Filter 1: Age

To match the DMDD DSM-5 criteria of filter 2 (age) i.e. the diagnosis should not be made for the first time before age 6 years or after age 18 years, subject’s date of birth and date of testing was used to calculate age at screening.

#### Filter 2: Symptoms (severity and frequency)

To fulfil the diagnostic DMDD DSM-5 criteria for filter 2 [symptoms (severity and frequency)] (A) severe recurrent temper outbursts; (B) the temper outbursts are inconsistent with developmental level; (C) the temper outburst occurs three or more times per week and (D) the mood between temper outbursts is persistently irritable or angry for most of the time and is noticed by others such as parents and teachers were used. To match these criteria the following items from KSADS was used:

- Irritability and anger—current summary score (KSADS Baseline—Bipolar Screen)
- Irritability and anger—current summary score (KSADS Baseline—Depression Screen)
- Loses temper—current summary score (KSADS Baseline—Other Disorders Screen)
- Initiate fights—current summary score (KSADS Baseline—Other Disorders Screen)
- Easily annoyed or angered—current summary score (KSADS Baseline—Behavioural Disorders Supplement)
- Vandalism—current summary score (KSADS Baseline—Behavioural Disorders Supplement)
- Physical cruelty to persons—current summary score (KSADS Baseline—Behavioural Disorders Supplement).

#### Filter 3: Observable by others

To match the DMDD DSM-5 criteria of filter 3 (observed by others) i.e. the mood between temper outbursts is persistently irritable or angry most of the day, nearly every day, and is observable by others (e.g. parents, teachers peers), items from the Parent reported KSADS were used:

- Irritability and anger—current parent score (KSADS Baseline—Bipolar Screen)
- Irritability and anger—current parent score (KSADS Baseline—Depression Screen)
- Loses temper—current parent score (KSADS Baseline—Other Disorders Screen)
- Initiate fights—current parent score (KSADS Baseline—Other Disorders Screen)
- Easily annoyed or angered—current parent score (KSADS Baseline—Behavioural Disorders Supplement)
- Vandalism—current parent score (KSADS Baseline—Behavioural Disorders Supplement)
- Physical cruelty to persons—current parent score (KSADS Baseline—Behavioural Disorders Supplement).

#### Filter 4: Pervasiveness/Setting

In order match the DMDD DSM-5 criteria of filter 4 (pervasiveness/settings), i.e. criteria F, where criterion A–D are present in at least two of three settings (i.e. home, at school, with peers), the following items from the KSADS were used:

- ODD impairment summary scores for current symptoms (KSADS Baseline—Behavioural Disorders Supplement)
- CD impairment summary scores for current symptoms (KSADS Baseline—Behavioural Disorders Supplement)
- Mood lability impairment summary scores for current symptoms (KSADS Baseline—Bipolar Screen).

#### Filter 5: Duration

To match the DMDD DSM-5 criteria of filter 5 (duration) i.e. criteria A and D have been present for 12 or more months, the age at testing and KSADS was used:

- Age at testing if below ages of 11
- ADHD symptoms occurring before the age of 7 (KSADS Baseline—Behavioural Disorder Supplement)
- ODD/CD symptoms occurring before the age of 10 (KSADS Baseline—Behavioural Disorder Supplement).

#### Filter 6: Exclusion due to comorbidity

To match the DMDD DSM-5 criteria of filter 5 (exclusion due to comorbidity), the KSADS Bipolar, Depression, Post-Traumatic Stress Disorder (PTSD), Anxiety Disorders, and Pervasive Developmental Disorders (PDD) supplements were used:

- KSADS Bipolar Screen: Single Manic Episode DSM-IV-TR criteria current
- KSADS Bipolar Screen: Single Hypomanic Episode DSM-IV-TR criteria current
- KSADS Bipolar Screen: Bipolar Disorder DSM-IV-TR criteria current
- KSADS PDD Supplement: Autistic Disorder DSM-IV-TR criteria current
- KSADS PDD Supplement: Asperger’s Disorder DSM-IV-TR criteria current
- KSADS PDD Supplement: PDD NOS Disorder DSM-IV-TR criteria current
- KSADS PTSD Screen: PTSD DSM-IV-TR criteria current
- KSADS Anxiety Supplement: Separation Anxiety DSM-IV-TR criteria current
- KSADS Anxiety Supplement: Panic Disorder DSM-IV-TR criteria current
- KSADS Anxiety Supplement: Phobic Disorder DSM-IV-TR criteria current
- KSADS Anxiety Supplement: Generalised Anxiety Disorder DSM-IV-TR criteria current
- KSADS Anxiety Supplement: Obsessive–Compulsive Disorder DSM-IV-TR criteria current
- KSADS Depression Screen: Dysthymia DSM-IV-TR criteria current.

### Appendix B: MDEC filters

#### Filter 1: Age

Despite the ICD-10 for MDEC not specifying an age criteria as stated in the DSM-5, the age criteria ranging from 6 years of age to 18 years was utilised, as this was the age inclusion criteria for the initial study sample.

#### Filter 2: Symptoms

To fulfil the diagnostic MDEC ICD-10 criteria for filter 2 (symptoms [severity and frequency]) persistently aggressive, dissocial or defiant behaviour with overt and marked symptoms of depression, anxiety or other emotional upsets. The criteria for both conduct disorders of childhood (F9l.-) and emotional disorders of childhood (F93.-) or an adult-type neurotic diagnosis (F40–F48) or a mood disorder (F30–F39) must be met. To match these criteria, filter 2 was split into 2a (i.e. to match conduct disorder criteria first) and 2b (i.e. to match emotional disorders of childhood, or adult neurotic diagnosis). In order to define filter 2a the following items were used from the KSADS:

- Loses temper—current summary score (KSADS Baseline—ODD Screen)
- Argues a lot with adults—current summary score (KSADS Baseline—Behavioural ODD Screen)
- Disobeys rules a lot—current summary score (KSADS Baseline—ODD Screen)
- Easily annoyed or angered—current summary score (KSADS Baseline—Behavioural Disorders Supplement)
- Angry or resentful—current summary score (KSADS Baseline—Behavioural Disorders Supplement)
- Spiteful and vindictive—current summary score (KSADS Baseline—Behavioural Disorders Supplement)
- Annoys people on purpose—current summary score (KSADS Baseline—Behavioural Disorders Supplement)
- Blames others for own mistakes—current summary score (KSADS Baseline—Behavioural Disorders Supplement)
- Lies—current summary score (KSADS Baseline—CD Screen)
- Truant—current summary score (KSADS Baseline—CD Screen)
- Initiates physical fights—current summary score (KSADS—CD Screen)
- Bullies threatens or intimidates others—current summary score (KSADS—CD Screen)
- Nonaggressive stealing—current summary score (KSADS—CD Screen)
- Vandalism—current summary score (KSADS Baseline—Behavioural Disorders Supplement)
- Breaking and entering—current summary score (KSADS Baseline—Behavioural Disorders Supplement)
- Aggressive stealing—current summary score (KSADS Baseline—Behavioural Disorders Supplement)
- Fire Starting—current summary score (KSADS Baseline—Behavioural Disorders Supplement)
- Often stays out at night—current summary score (KSADS Baseline—Behavioural Disorders Supplement)
- Ran away overnight—current summary score (KSADS Baseline—Behavioural Disorders Supplement)
- Use of weapon—current summary score (KSADS Baseline—Behavioural Disorders Supplement)
- Physical cruelty to persons—current summary score (KSADS Baseline—Behavioural Disorders Supplement)
- Forced sexual activity—current summary score (KSADS Baseline—Behavioural Disorders Supplement)
- Cruelty to animals—current summary score (KSADS Baseline—Behavioural Disorders Supplement).

When highlighting additional symptoms relating to an emotional disorders of childhood (F93.-) or an adult-type neurotic diagnosis (F40–F48) or a mood disorder (F30–F39) the following items were used, denoted as filter 2b:

- Elation, expansive mood—current summary score (KSADS—Bipolar Screen)
- Mood lability—current summary score (KSADS—Bipolar Screen)
- Depressed mood—current summary score (KSADS—Depression Screen)
- Irritability and anger—current summary score (KSADS—Depression Screen)
- Anhedonia—current summary score (KSADS—Depression Screen)
- Panic attacks—current summary score (KSADS—Panic Disorder Screen)
- Fears calamitous event will cause separation—current summary score (KSADS—Separation Anxiety Screen)
- Fears harm befalling attachment figure—current summary score (KSADS—Separation Anxiety Screen)
- School reluctance/refusal—current summary score (KSADS—Separation Anxiety Screen)
- Fears sleeping alone—current summary score (KSADS—Separation Anxiety Screen)
- Fears being at home alone—current summary score (KSADS—Separation Anxiety Screen)
- Shrinks from contact—current summary score (KSADS—Social Anxiety Screen)
- Fear of social situations—current summary score (KSADS—Social Anxiety Screen)
- Agoraphobia distress—current summary score (KSADS—Agoraphobia Screen)
- Agoraphobia avoidance—current summary score (KSADS—Agoraphobia Screen)
- Unrealistic worry about future—current summary score (KSADS—GAD Screen)
- Somatic complaints—current summary score (KSADS—GAD Screen)
- Marked self-consciousness—current summary score (KSADS—GAD Screen)
- Marked feeling of tension, unable to relax—current summary score (KSADS—GAD Screen)
- OCD compulsions—current summary score (KSADS—OCD Screen)
- OCD obsessions—current summary score (KSADS—OCD Screen).

In order to pass threshold for filter 2, individuals must reach threshold for at least one filter in 2a and one filter in 2b.

#### Filter 3: Duration

To match the MDED ICD-10 criteria of filter 4 (duration) i.e. the criteria for both conduct disorders of childhood (F9l.-) must be met, where these symptoms must be an enduring pattern of behaviour lasting 6 months or longer, the KSADS current duration identifying as to whether symptoms have been present for the past 6 months was used. In order to meet the duration criteria for the mixed emotions feature present within MDEC, current depression raw scores from the three core symptoms within the screening scores were used as they capture any difficulties over the past 2 weeks. With reference to anxiety disorders, the duration criteria capturing symptom difficulties over the past 6 months was implemented. Specific items for the duration criteria are listed below:

- CD duration current summary score (KSADS Baseline—Behavioural Disorders Supplement)
- ODD duration current summary score (KSADS Baseline—Behavioural Disorders Supplement)
- Depressed Mood threshold of 4 or above current summary score (LAMS UK KSADS Baseline—Depression Screening)
- Irritability and Anger threshold of 4 or above current summary score (LAMS UK KSADS Baseline—Depression Screening)
- Anhedonia threshold of 3 or above current summary score (LAMS UK KSADS Baseline—Depression Screening)
- Separation Anxiety duration summary of current episode (LAMS UK KSADS Baseline—Anxiety Disorders Supplement)
- Specific Phobia duration summary of current episode (LAMS UK KSADS Baseline—Anxiety Disorders Supplement)
- GAD duration summary of current episode ((LAMS UK KSADS Baseline—Anxiety Disorders Supplement).

#### Filter 4: Pervasiveness/setting

In order match the MDEC ICD-10 criteria of filter 5 (pervasiveness/settings), the ICD-10 highlights that the child must meet diagnostic threshold for the combination of persistently aggressive, dissocial or defiant behaviour with overt and marked symptoms of depression, anxiety or other emotional upsets, and that the criteria for conduct disorder (F91) must also be meet including repetitive and persistent patterns of such behaviours. In order to address the pervasiveness criteria for MDEC the KSADS ‘Impairment’ screening criteria across social, family and school settings for Depression Scores and both the Anxiety Disorders and Behavioural Disorders Supplements was used.

- ODD impairment summary scores for current symptoms (LAMS UK KSADS Baseline—Behavioural Disorders Supplement)
- CD impairment summary scores for current symptoms (LAMS UK KSADS Baseline—Behavioural Disorders Supplement)
- Depression impairment summary scores for current symptoms (LAMS UK KSADS Baseline—Depression Screening)
- Panic Disorder impairment summary scores for current symptoms (LAMS UK KSADS Baseline—Anxiety Disorders Supplement)
- Separation Anxiety Disorder impairment summary scores for current symptoms (LAMS UK KSADS Baseline—Anxiety Disorders Supplement)
- Phobic Disorder impairment summary scores for current symptoms (LAMS UK KSADS Baseline—Anxiety Disorders Supplement)
- Overanxious Generalised Anxiety Disorder impairment summary scores for current symptoms (LAMS UK KSADS Baseline—Anxiety Disorders Supplement)
- Obsessive Compulsive Disorder—Compulsions impairment summary scores for current symptoms (LAMS UK KSADS Baseline—Anxiety Disorders Supplement)
- Obsessive Compulsive Disorder—Obsessions impairment summary scores for current symptoms (LAMS UK KSADS Baseline—Anxiety Disorders Supplement).

#### Filter 5: Impairment

In order to address the impairment of MDEC the last items i.e. ‘How often do the behaviours in category *X* interfere with your child’s ability to do schoolwork or get along with other people?’ from the Parent report of the Child and Adolescent Symptoms Inventory (CASSI-4) from categories B, C, D, E, G, H, I and K were used:

- Item Bx: ODD impairment (CAASI—Baseline Parent)
- Item Cx: CD Impairment (CAASI—Baseline Parent)
- Item Dx: Worries (CAASI—Baseline Parent)
- Item Ex: Other Anxiety (CAASI—Baseline Parent)
- Item Gx: Separation Anxiety (CAASI—Baseline Parent)
- Item Hx: Other disorders of social communication and conduct (CAASI—Baseline Parent)
- Item Ix: Enuresis (CAASI—Baseline Parent)
- Item Kx: Depression (CAASI—Baseline Parent).

#### Filter 6: Comorbidity/exclusion criteria

To match the MDEC ICD-10 criteria for filter 7 (exclusion due to comorbidity), i.e. diagnosis of conduct disorder cannot be given for dissocial personality disorder (F60.2), schizophrenia (F20.-), manic episode (F30.-), depressive episode (F32.-), pervasive developmental disorder (F84.-), or hyperkinetic disorder (F90.-), items from the KSADS were used:

- KSADS Psychotic Disorders Supplement: Schizophrenia DSM-IV-TR criteria current
- KSADS Bipolar Screen: Single manic Episode DSM-IV-TR criteria current
- KSADS Depression Screen: Depressive Episode DSM-IV-TR criteria current
- KSADS PDD Supplement: Autistic Disorder DSM-IV-TR criteria current
- KSADS PDD Supplement: Asperger’s Disorder DSM-IV-TR criteria current
- KSADS PDD Supplement: PDD NOS Disorder DSM-IV-TR criteria current.

## Abbreviations

BD: Bipolar Disorder
CD: Conduct Disorder
DMDD: Disruptive Mood Dysregulation Disorder
DSM-5: Diagnostic and Statistical Manual of Mental Disorders-5
ICD-10: International Classification of Diseases-10
LAMS: Longitudinal Assessment of Manic Symptoms
MDEC: Mixed Disorder of Emotions and Conduct
ODD: Oppositional Defiant Disorder
PBD: Paediatric Bipolar Disorder
WHO: World Health Organisation
